# The 3rd DBCLS BioHackathon: improving life science data integration with Semantic Web technologies

**DOI:** 10.1186/2041-1480-4-6

**Published:** 2013-02-11

**Authors:** Toshiaki Katayama, Mark D Wilkinson, Gos Micklem, Shuichi Kawashima, Atsuko Yamaguchi, Mitsuteru Nakao, Yasunori Yamamoto, Shinobu Okamoto, Kenta Oouchida, Hong-Woo Chun, Jan Aerts, Hammad Afzal, Erick Antezana, Kazuharu Arakawa, Bruno Aranda, Francois Belleau, Jerven Bolleman, Raoul JP Bonnal, Brad Chapman, Peter JA Cock, Tore Eriksson, Paul MK Gordon, Naohisa Goto, Kazuhiro Hayashi, Heiko Horn, Ryosuke Ishiwata, Eli Kaminuma, Arek Kasprzyk, Hideya Kawaji, Nobuhiro Kido, Young Joo Kim, Akira R Kinjo, Fumikazu Konishi, Kyung-Hoon Kwon, Alberto Labarga, Anna-Lena Lamprecht, Yu Lin, Pierre Lindenbaum, Luke McCarthy, Hideyuki Morita, Katsuhiko Murakami, Koji Nagao, Kozo Nishida, Kunihiro Nishimura, Tatsuya Nishizawa, Soichi Ogishima, Keiichiro Ono, Kazuki Oshita, Keun-Joon Park, Pjotr Prins, Taro L Saito, Matthias Samwald, Venkata P Satagopam, Yasumasa Shigemoto, Richard Smith, Andrea Splendiani, Hideaki Sugawara, James Taylor, Rutger A Vos, David Withers, Chisato Yamasaki, Christian M Zmasek, Shoko Kawamoto, Kosaku Okubo, Kiyoshi Asai, Toshihisa Takagi

**Affiliations:** 1 Database Center for Life Science, Research Organization of Information and Systems, 2-11-16, Yayoi, Bunkyo-ku, Tokyo, 113-0032, Japan

**Keywords:** BioHackathon, Open source, Software, Semantic Web, Databases, Data integration, Data visualization, Web services, Interfaces

## Abstract

**Background:**

BioHackathon 2010 was the third in a series of meetings hosted by the Database Center for Life Sciences (DBCLS) in Tokyo, Japan. The overall goal of the BioHackathon series is to improve the quality and accessibility of life science research data on the Web by bringing together representatives from public databases, analytical tool providers, and cyber-infrastructure researchers to jointly tackle important challenges in the area of *in silico* biological research.

**Results:**

The theme of BioHackathon 2010 was the 'Semantic Web', and all attendees gathered with the shared goal of producing Semantic Web data from their respective resources, and/or consuming or interacting those data using their tools and interfaces. We discussed on topics including guidelines for designing semantic data and interoperability of resources. We consequently developed tools and clients for analysis and visualization.

**Conclusion:**

We provide a meeting report from BioHackathon 2010, in which we describe the discussions, decisions, and breakthroughs made as we moved towards compliance with Semantic Web technologies - from source provider, through middleware, to the end-consumer.

## Background

The term Semantic Web refers to those parts of the World Wide Web in which information is explicitly encoded in a machine-readable syntax, and the relationships between entities are explicitly constructed and labeled using machine-readable links. The most significant difference between the Semantic Web and the current World Wide Web is that Semantic Web is intended to be accessed by machines, rather than by people. As such, it concerns itself primarily with the structured representation of data and knowledge in ways that can be automatically interpreted and traversed without human intervention. This should therefore support more complex, cross-domain, and cross-resource investigations by putting the burden of data discovery and integration on the machine, rather than on the individual.

Until now, complex cross-domain querying has tended to only be supported by large-scale data warehouses, including BioMart [1] and InterMine [2]. However, these can only respond to questions within the confines of the data collected in that warehouse. By simplifying and automating dynamic and distributed data discovery and integration, the Semantic Web should encourage a researcher’s curiosity and support them in pursuing “spontaneous” questions outside of the scope of existing and pre-constructed data warehouses.

Early-adopters of Semantic Web technologies have put together demonstrations showing its power over traditional data and knowledge frameworks. Among the most prominent of these early-adopters have been the life and health science communities [3] where numerous Linked Data initiatives have emerged. Notable examples include the Semantic Web Healthcare and Life Science Interest Group (HCLSIG) with their creation of the Clinical Observations Interoperability demo [4] and Linked Open Drug Data demo [5]; and the Bio2RDF project [6,7], which integrates more than 50 biological databases into a Linked Data environment. While these motivational projects demonstrate the power of Linked Data [8], the warehouse approach they adopt, which ensures data consistency, avoids the desirable and intended distributed nature of the Semantic Web. The next step in the Semantic Web evolution involves the source providers themselves making their resources available as Linked Data. This was the theme of BioHackathon 2010 - to our knowledge the first time in the life sciences that such a large group of non-affiliated data providers, cyber-infrastructure projects, and client-application projects have come together for this purpose.

We use the terms “Linked Data” and “Semantic Web” to refer to two different aspects of the construction of a distributed web of data and knowledge, and it is useful to go into some detail about how each layer contributes to the overall vision of machine-readable data and knowledge.

Linked Data refers to data exposed on the Web following a particular set of technologies and conventions aimed at improving mechanized access and processing. For example, in Linked Data, Uniform Resource Identifiers (URIs) are used to refer to all data entities; moreover, by convention and best practice, these URIs should (in general) resolve to additional Linked Data describing that entity, and linking that entity’s URI to other URIs using explicitly labeled relationships. These Linked Data conventions provide significant advantages for both data providers and data consumers. First, through naming data by a URI, the means for retrieving that data, regardless of its provider or location, become uniform - defined by the HTTP protocol. Similarly, there is a separation of how data is stored (web pages, flat-files, relational databases, etc.) and how it is accessed and consumed. In much the same way as the HTTP protocol created a uniform access layer for the Web, allowing the creation of generic tools such as Web browsers, Linked Data ensures that, regardless of underlying format, exposed information is uniformly accessible through a common query language - SPARQL. More importantly, Linked Data can also be integrated site-to-site across multiple independent providers via queries that span multiple data-endpoints. Linked Data technologies and conventions, therefore, facilitate data exploration and evaluation by removing the need to design an integrative schema, download, homogenize, and finally warehouse data subsets in order to ask common domain-spanning questions.

The Semantic Web extends the concept of and is built on Linked Data, but is additionally concerned with defining machine-interpretable semantics for entities and relations that might appear in a Linked Dataset. The precise meanings of these concepts and relations are defined in an ontology, and this ontology can be utilized by software called a reasoner to evaluate the data-types and properties within a Linked Dataset against a possible interpretation of that dataset, defined by the ontology. Through this process, the aggregated data is automatically classified or categorized according to the concepts defined by a given ontology - the ontology provides a “view” of the data, and different ontologies can be used to provide differing views, depending on the nature of the study or the question of interest. Moreover, new knowledge can be automatically derived as reasoners detect new instances of ontologically-defined concepts within aggregated Linked Data.

For both biological researchers and data managers, the significance of these new paradigms cannot be over-stated. Laboratories that currently invest significant resources in creating one-off integrated data warehouses, and then manually interpreting them, would be able to create such datasets with a single Web-wide query. Moreover, carefully crafted ontologies could then be employed to automatically classify the resulting integrated data into conceptual categories of interest to that researcher. Beyond keyword searches, or even traditional database queries, Semantic Web technologies facilitate querying datasets at a conceptual level, where the concepts of interest can be defined post-facto, after the act of data query and integration. The ease and low-cost of creating and analyzing such datasets would encourage researchers to re-ask their questions in light of new discoveries or new datasets. Alternately, they may choose to re-formulate their research question as they progress toward a new discovery, in the knowledge that retrieving and integrating additional third-party data into their existing warehouse can be achieved with very little cost. While these kinds of integrative investigations can be (and are being) conducted on the current Web, the current cost and complexity of cross-domain data integration hinders exploration, inhibiting researchers from pursuing their own curiosities.

The goal of the 2010 BioHackathon, therefore, was to bring together data providers and tool authors to discuss the path toward making their resources available using these powerful new integrative standards and frameworks.

### Meeting outline

A total of 56 attendees participated in BioHackathon 2010, including representatives from at least 10 major biological databases, major cyber-infrastructure projects such as BioRuby [9,10], Biopython [11,12], SADI [13,14], G-language [15,16], BioMart and InterMine, and client providers such as Taverna [17], FlyMine [18,19], Bio-jETI [20,21], and Cytoscape [22,23]. The full list of BioHackathon participants, and their respective projects, is available [24].

Many important life science data providers were present at the BioHackathon, including UniProt [25,26], Korean HapMap [27], TreeBASE [28], DDBJ [29,30], INSD [30], PDBj [31], KEGG [32,33], DBCLS [34], IntAct [35,36], as well as developers of data integration projects including Bio2RDF [6,7], BioGateway [37,38], DERI [39], PSICQUIC [40,41] and the HUPO Proteomics Standards Initiative [42].

**Figure 1:**
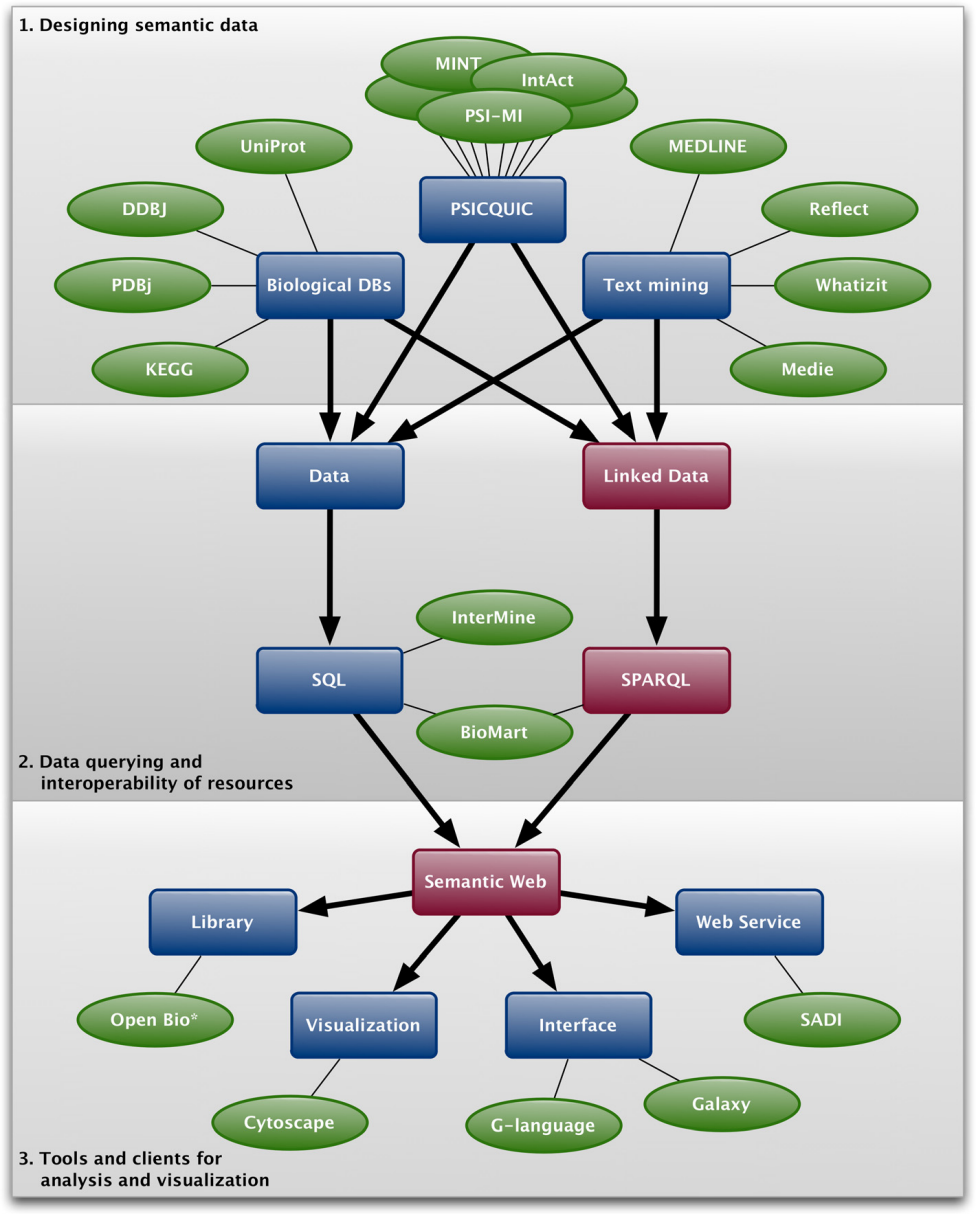
**A schematic flow from data through querying to analysis, visualization and browsing that were collaboratively endeavored in the BioHackathon 2010.** Databases, services, projects represented and/or utilized are shown in green ovals.

BioHackathon 2010 revolved around discussion and hands-on exploration of approaches to structuring life science Linked Data such that it maximizes the power of semantics, while at the same time minimizing the burden placed on data providers. The lessons learned and best practices that emerged from these discussions are detailed below and follow the flow of Figure 1 from designing data through querying to analysis, visualization and browsing.

## Methods

### Ontologies

Ontologies and ontology-like knowledge structures have been part of life science research and practice for centuries. For example, both Merriam-Webster and the Oxford English dictionary suggest that the first use of the term “nosology” (a formal classification of diseases) occurred in approximately 1721, and of course formal property-based taxonomies are almost as old as biology itself. Medical and clinical terminologies such as SNOMED CT [43] and OpenGALEN [44] have been developed over the past decades. However, the term “ontology”, in its more modern usage in the life sciences, arguably began with the establishment of the Gene Ontology project in 1998 [45] which aimed to set-up a classification system for genes. Subsequently, the idea of using ontologies to categorize and annotate all types of life science data rapidly has become widely adopted. To encompass all of the potential types and uses for ontologies, some very generalized definitions of “ontology” have been proposed, with perhaps the most widely cited being “a specification of a conceptualization” [46] - quite simply, to be formal and explicit about the “things”, concepts and relationships that exist in a domain of interest. The Artificial Intelligence community further specified this definition for their own field, as “the models that capture and describe specific domains” [47].

At the intersection of the life sciences and the computer sciences, we find ontologies being used for a wide variety of purposes, such as annotation (e.g., plant anatomy [48]), supporting biological analysis (e.g., systems biology modeling [49]); data integration and improved shareability (e.g., the Gene Ontology [45]); and decision support (e.g., Clinical Practice Guidelines [50]).

Given the wide range of use-cases and disparate development communities, a variety of standards and structures emerged within which to capture this explicit ontological knowledge. The Semantic Web initiative of the World Wide Web Consortium (W3C) consolidated these into three core standards for data representation, knowledge representation, and querying, described as follows.

### RDF

The Resource Description Framework (RDF) is a data model proposed by the W3C to implement and support the Semantic Web infrastructure. RDF consists of three components - Resources, Relationships, and Literals. A Resource is any nameable “thing” (e.g., an entity or a concept) and RDF Resources are always named by Universal Resource Indicators (URIs). Optimally, every Resource should only have one URI that is shared throughout the Web, though in practice this is quite difficult to achieve (and, in fact, was a topic of significant discussion at the BioHackathon, as will be described below). The second component is the Relationship, also called the “Predicate”. Relationships are used to describe how two Resources are related to one another; these are also named by a URI, and this named relationship should also be optimally unique and shared throughout the Web. The combination of two Resources (*subject* and *object*) and their Relationship (*predicate*) is called a “Triple” - the smallest unit of information that can be represented on the Semantic Web. For example:

<http://example.org/event/BH10> <http://example.org/attendee> <http://example.org/people/TK>

The final component is the Literal - effectively, a numerical value or a set of characters. Literals are intended to provide concrete data about a particular Resource. As with Resource-to-Resource connections, Literals are connected to a Resource by an appropriate and well-defined Relationship URI, for example:

<http://example.org/event/BH10>  <http://example.org/year> “2010”

Literals cannot be the *subject* of a Triple, and therefore cannot be connected to one another. A set of Triples is called a “Graph”, and Triples are generally stored in a database-like “triple-store”. These databases can then be exposed on the Web as “endpoints” available for querying by a variety of tools.

RDF can be represented in various ways for the purpose of passing data from one machine to another, or for human consumption. One of the most common representations is XML [51]. Another common serialization is N3 [52], which is much more compact.

### OWL

The second of the core W3C Semantic Web standards is the Web Ontology Language (OWL). OWL is a language for encoding (a) how Classes and Predicates should be interpreted, and (b) how specific combinations of Resources and Predicates can be inferred to represent a particular concept. For example, the concept of (putative) “TransmembraneProtein” might be simplistically defined in pseudo-OWL as follows:

TransmembraneProtein is: a:Protein located_in a:Membrane and has_sequence (a:Sequence and [has_motif a:Helix or a:Barrel])

 Subsequently, if the following triples were found on the Web:

1. ex:molecule type a:Protein

2. ex:molecule has_sequence ex:sequence

3. ex:sequence has_motif a:Helix

4. ex:molecule located_in a:Membrane

It would be possible for a reasoner (a program that analyses the logical statements in RDF and OWL) to conclude that ex:molecule is of ontologically-defined type “TransmembraneProtein”.

What may not be obvious from this example is that triples 1–4 might come from entirely different places on the Web. However, it is possible that triple 3 is brought from bioinformatics analysis and triple 4 is acquired experimentally. Because they are sharing URIs, the independently-derived triples can be easily combined into a Graph. Moreover, OWL and reasoning can then be applied to interpret (“discover”) the emergent new information contained in that integrated dataset. This idea is extremely powerful. However, to achieve this power, consensus must be reached on how to represent the data in RDF such that it can be integrated as easily as just described, and this was a major theme of the BioHackathon.

### SPARQL

SPARQL (SPARQL Protocol and RDF Query Language) [53] is a standard language for querying RDF data, allowing the information stored in triple stores to be explored and retrieved in a manner akin to how SQL is used to retrieve data from relational databases. A triple store that is queryable by the SPARQL language is referred to as a “SPARQL endpoint”. The life science community consisted of early adopters of this technology, providing SPARQL endpoints even before this language became an official W3C recommendation [37,38]. SPARQL queries consist of a series of triple-patterns, where any component of the triple might be a variable, and these triple-patterns can be combined into graph-patterns. SPARQL engines then look for “sub-graphs” that match the graph-pattern specified in the query. For example, the triple-pattern:

?protein <http://www.semantic-systems-biology.org/SSB#located_in> "nuclear membrane"

could be used to find all of the proteins in a given SPARQL endpoint that are located in the nuclear membrane.

To help construct SPARQL queries, efforts to bring these technologies closer to end-users are emerging, providing straightforward interfaces to domain-specific triple stores (e.g., Cell Cycle Ontology [54]).

## Results

### Designing semantic data

A major focus of the BioHackathon was to look at RDF from the data provider's perspective. We discussed, and in some cases came-up with possible answers to, questions such as “What Semantic Web-enabled life science data should be provided?”, “Should I convert everything into RDF, or just some types of data?”, “What data is already available as RDF and how do I link into that?”, “I am a publisher of RDF already, how might I improve my current offerings?” This section, therefore, first describes a number of guidelines that were established at the BioHackathon, then examines the state of currently available data, and concludes with the open questions that remained at the end of the BioHackathon.

The guidelines center around two issues: making data available in ways that are easy to integrate and query, and making data descriptive and explicit. Addressing the former, the participants agreed that providers should ideally both:

• provide a SPARQL endpoint, not necessarily hosted by the data provider themselves, but officially supported by the provider, and

• make dumps of raw data available in RDF from their HTTP or FTP server.

Further, the BioHackathon attendees agreed that in order to provide descriptive and explicit data, providers should strive to:

• use standard/shared URIs, and

• use standard/shared predicates.

UniProt is an example of an existing provider who is following all of these guidelines. It offers RDF dumps on their FTP server [55], and also allows dynamic retrieval of RDF representations of specific resources (via adding '.rdf' to the URL or via HTTP content negotiation) and/or query results (via appending '&format=rdf'). TreeBASE does the latter as well, and can make an RDF dump available easily. Representatives from DDBJ provided the Gene Trek in Prokaryote Space (GTPS) [56] data as an RDF dump and are also working to convert their other resources into RDF. PDBj made available their entire structure database in the RDF format [57]. The KEGG group also attempted to convert their data into RDF but the work is temporally discontinued due to the recent change of their licensing policy. TogoWS [58,59] has implemented on-the-fly RDF conversion for databases stored in TogoDB [60] and several external data sources including NCBI PubMed (e.g., http://togows.dbcls.jp/entry/pubmed/20472643.ttl).

Even though the size of the RDF representation of data can generally become larger than the original one, users can benefit from advanced-level search capabilities based on the semantics explicitly embedded in the RDF. Therefore, providers agreed to make RDF dumps available for download. However, providing a SPARQL endpoint requires additional time and resources, and these will likely take longer to appear from the individual data hosts. There are a number of SPARQL endpoints provided by third-parties, many of which are warehouses of RDF data from these individual providers. Examples include the LODD [5] and the Bio2RDF [6,7]; however, the RDF in these warehouses generally is a project-specific conversion from the source data host, and may not resemble RDF provided by the host itself, if such is available. Finally, though many are overlapping in their scope, these third-party warehouse projects have created independent models for the RDF data, and independent standards for URIs, so are difficult to integrate with one another. The problem of URI standardization was discussed extensively at the BioHackathon, and is the next topic of discussion here.

### URIs and global integration

The URI is the core technology upon which all aspects of the Semantic Web are built. Thus, as with all Semantic Web projects, it was inevitable that the first decision that needed to be made at BioHackathon 2010 was related to URIs. The BioHackathon attempted to use a community-consensus approach to find a solution to generating commonly accepted URIs, and a consensus decision was achieved among the attendees in a surprisingly short time. The consensus is described as follows:

• The BioHackathon community recognized the need for, and strongly endorsed “Cool URIs” [61] as the behavior that they would strive to adhere to when naming their own entities. A “Cool URI” has a variety of behaviors, but most relevant to this meeting were that Cool URIs do not change, are precise in what they identified (i.e., is it the “real-world thing” or a document describing the “thing”), must resolve through HTTP GET, and can provide an appropriate representation of the “thing” through content-negotiation (e.g., HTML for a browser, and RDF for a Semantic Web tool). An example of a Cool URI is http://identifiers.org/obo.go/GO:0006915, which can be resolved to an RDF representation with the “Accept application rdf+xml” HTTP header, or resolved to an HTML representation with the “Accept text/html” HTTP header.

• It was recognized that, in order to link data, it is often necessary to refer to the entities of third-parties using common URIs, and that, for the time being, many of these third-party providers do not have “Cool URIs” for the entities we wish to cross-reference, but it is important to be “polite” [62] when referring to their data in our RDF. Effectively, when a data provider publishes a URI, he/she does so expecting people to use that URI in their own RDF Triples, so if he/she is publishing a URI about someone else’s data (i.e. he/she is naming another’s data elements) then he/she should attempt to be “polite” about it.

What it means to be “polite” was discussed over several days, and the following recommendations were adopted by the attendees:

1. A registry of bioinformatics URI patterns has been set-up in FreeBase [63].

2. Native providers of Cool URIs should register their URI pattern in FreeBase, with an indication that it is “approved” for use by third-parties, and has some guarantee of stability/resolvability in the long-term.

3. A provider who must refer to a third-party entity in their RDF, should first check FreeBase to determine which URI pattern has been registered there.

4. If they find no existing entry for that provider, they should attempt to determine which URI pattern from that provider is most likely to be stable (giving preference to Cool-URI-style URIs over GET strings with parameters) and then register that pattern in the FreeBase repository. This is the pattern that will then be used by the rest of the community until such time as the third-party provides Cool-URI-style identifiers.

5. When such third-parties do begin to produce Cool-URIs, they are strongly encouraged to register a mapping scheme to assist the community in accurately translating from the URIs they have been using in their RDF to the URIs that are now approved by that third party.

This first-come-first-served approach is intended to be purely pragmatic. It ensures that nobody is blocked from making progress on their own data representation by the need for community consensus around third-party URI patterns, and is highly scalable by distributing the curatorial burden over the entire community in a needs-based manner. However, at the time of this writing, this proposal has not been implemented even by BioHackathon attendees, suggesting that this solution was impractical, undesirable, or both. As such, referring to and linking to third-party data in RDF remains a challenge for many providers.

While there was some discussion regarding exactly how to structure the RDF returned by a URI when dereferenced, there was general agreement that this discussion was better left to a future meeting. Two providers of Semantic Web data - the Life Science Resource Names (LSRN [64] - soon to be supplanted by Identifiers.org [65]) and SADI projects - have decided to adopt the Semanticscience Integrated Ontology (SIO) [66] to model at least core metadata about the nature of the URI that has been resolved. SIO is an ontology under active development specifically aimed at describing scientific data and data-set-composition, for example, the type of “thing” being described (a GenBank record, a SwissProt record, etc.) and/or the dataset to which it belongs (spot information from a particular microarray study, for example).

### Data providers

The success of any Semantic Web initiative in the life sciences will depend on the participation of the source data providers. As such, their involvement in the BioHackathon was key to many of the other activities undertaken during the event. Here we describe some of the successes achieved by the various domains of bio/molecular data resource providers.

#### Molecular databases

Participants representing molecular database providers indicated that they expected to produce their public resources officially in the RDF format in the near future. As mentioned earlier, UniProt already provides the raw data in RDF. During the BioHackathon, the DDBJ group developed a prototype of a converter which returns the RDF version of a DNA sequence entry; however, because the International Nucleotide Sequence Database Collaboration (INSDC) data format is very complicated, there is a need to improve the design of the RDF representation to maximize usability. The KEGG group attempted to extract cross-referencing information between pathways, genes, and Protein Data Bank (PDB) entries and to capture these in RDF. In addition, the PDBj group developed an Extensible Stylesheet Language (XSL) that converted the entirety of the PDB Markup Language (PDBML) file into RDF. Since the PDBML is based on the macromolecular Crystallographic Information File (mmCIF) format - the original format of the PDB database - and mmCIF itself is defined as an ontology, mmCIF categories and items are easily re-used as predicates in an RDF representation of PDBML [57].

#### Molecular interactions

The Proteomics Standard Initiative Common QUery InterfaCe (PSICQUIC) project [40,41] is moving towards an RDF/XML output format. PSICQUIC is a standard that defines a common way to access Molecular Interaction resources (IntAct [35,36], MINT [67], BioGrid [68], iRefIndex [69], MatrixDB [70], Reactome [71], MPIDB [72], ChEMBL [73]), including more than 1.7 million interactions in 2010/2011, and at the time of this writing this number had grown to 150 million interactions, spanning 27 PSICQUIC services over 19 independent organizations [74]. This rapid and widespread adoption was due in large part to the notable successes of the proteomics community in coordinating their data sharing efforts via PSICQUIC development over previous BioHackathon events, and we believe this is an excellent demonstration of the utility and power of creating communities of developers and providers through BioHackathon events.

**Figure 2:**
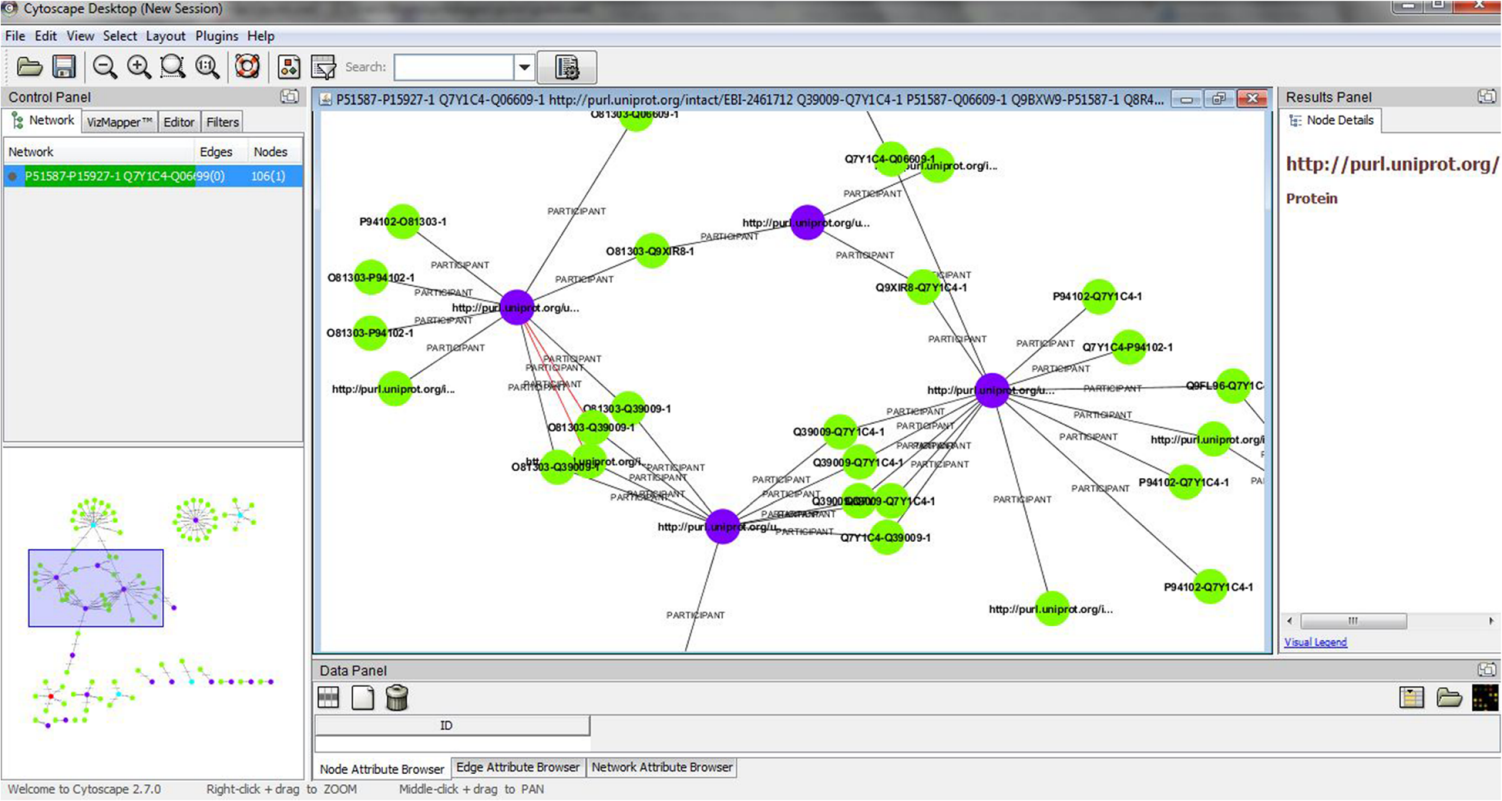
Visualizing PSICQUIC RDF output in Cytoscape.

The evolution of the standard that was enacted during BioHackathon 2010 was to design PSICQUIC services that return Biological Pathway Exchange (BioPAX) [75] Level 3 data. As the underlying data was in Proteomics Standards Initiative Molecular Interaction (PSI-MI XML) format [76], a converter was created using the Apache Jena framework [77], which is designed to simplify manipulation of RDF in Java. At the end of the BioHackathon, PSICQUIC was able to return RDF data in a variety of formats (RDF/XML, N3, and N-Triples). The data returned contains interaction data including information about the interaction participants and their individual cross-referencing information. To ensure data integrity and correct output formatting, the output was successfully visualized in Cytoscape 2.7.0 beta 3 (Figure 2).

#### Systems biology

In systems biology, most curated models are developed by a graphical tool (e.g., CellDesigner [78]) and described in Systems Biology Markup Language (SBML) format [79]. Each pathway model is published in a traditional manuscript, as well as a corresponding SBML model file. To reveal relationships among pathways and overall network structure, integration of those published models is required. For this purpose, BioPAX can be used as a common exchange format for biological pathway models. Users can use Semantic Web technologies to explore the integrated pathways since BioPAX data is represented in OWL.

To evaluate this approach in practice, we selected Alzheimer’s disease as a target domain, since representatives at the BioHackathon had already been involved in developing AlzPathway [80,81] which collects signaling and metabolic pathways relevant to this disease. Importantly, many pathways implicated to have a role in Alzheimer’s disease have other critical functions for normal cell and tissue growth and development. For example, the apoptosis pathway is known to be closely related to Alzheimer’s disease. Therefore, integration of apoptosis and AlzPathway models might bring novel insights and/or provide new hypotheses on a formerly unknown relationship between these pathways.

The sbml2biopax program [82] was used to convert the apoptosis pathway, which is published in BioPAX format as supplement material [83], into the SBML format. Both AlzPathway and apoptosis pathway data in BioPAX Level 2 format are stored in an instance of 4store [84] and SPARQL queries against the triple store were performed. The resulting integrated apoptosis and AlzPathway model was explored using RDF store and SPARQL queries. It was noted that mapping of names among models in RDF is also essential for successful integration in this SPARQL endpoint.

#### Taxonomy

Another application of Semantic Web technologies pursued at the BioHackathon was related to systematics and taxonomy [85]. Taxonomic concepts are at the heart of such well-established disciplines as zoology and botany and are crucial to the field of biodiversity informatics [86]. Recently they are becoming increasingly important in molecular biology and genomics as well, for example in the numerous metagenomics projects that deal with hundreds of taxonomic entities simultaneously (e.g., [87,88]) as well as the increased availability of genome data from non-traditional model organisms that becomes available for comparative-genomics studies. Simply representing a taxonomy as a string has a number of shortcomings. Chiefly among them is the inability to link to other data, the existence of synonyms (taxa with multiple names) and different taxa with the same name (homonyms). Using taxonomy-specific controlled vocabularies makes it possible to represent taxonomic information in the form of RDF triples and provides a means to link taxonomic names to other entities, such as molecular sequences, type collections and geographic information. Ontologies which have been designed for taxonomy and biodiversity informatics are the Taxonomic Database Working Group (TDWG) ontology [89] and DarwinCore [90]. Furthermore, the Comparative Data Analysis Ontology (CDAO) [91] is an ontology designed for the associated fields of evolutionary biology and phyloinformatics and enables the description of concepts related to comparative evolutionary analysis, including the analysis of molecular sequences. It is, however, worth noting that there is no authoritative source for taxonomy. This is mainly due to the fact that multiple needs get conflated. This issue has had a slight negative impact on semantic-based integrations so far (and usually an *ad-hoc* taxonomy is built based on extant resources).

#### Text-mining

At the present time, biological databases alone cannot capture the richness of scientific information and argumentation contained in the literature, nor can they provide support for the novel ways in which scientists wish to interrogate that data. A considerable fraction of the existing data in biology consists of natural language texts used to describe and communicate new discoveries, and therefore scientific papers constitute a resource with crucial importance for life sciences. As the amount of scholarly communication increases, it is increasingly difficult for specific core scientific statements to be found, connected, and curated.

The biomedical text mining community has been working for a long time on the development of reliable information extraction applications. Both named entity recognition and conceptual analysis are needed in order to map from natural language texts to a formal representation of the objects and concepts represented by the text, with direct links to online resources that explicitly expose those concepts as semantics.

Different Web tools allow researchers to search literature databases and integrate semantic information extracted from text with external databases and ontologies. At the time of the BioHackathon, Whatizit [92,93], Reflect [94-96] and Medie [97,98] provided Web APIs for free without any registration, and the developers and collaborators of the latter two services were members of this sub-group. Therefore, this BioHackathon sub-group concentrated on using these three tools to develop a methodology to provide RDF triples for PubMed literature. Further, we investigated how to embed annotations into the XHTML output from these tools, and selected RDFa (RDF in attributes [99]) a suitable technology for this. RDFa enables the embedding of RDF triples within XHTML documents, and also enables the extraction of RDF triples by compliant user-agents.

### Data querying and interoperability of resources

#### Implementation

The BioHackathon participants investigated many different facets of the existing Semantic Web technologies to help data provision and querying in the life sciences. Working with semantic data requires a variety of specialized software and libraries:

• Libraries for reading, transforming and writing RDF

• SPARQL servers (endpoints) for querying

• Semantic registries or indices enabling discovery and querying of data, e.g., SADI

• User interfaces and visualization tools such as RDFScape [100].

Although SPARQL offers the possibility of large-scale distributed flexible discovery and querying of data, it relies on widespread provision of RDF data through SPARQL endpoints. Realistically, it will take time to migrate existing resources to these new standards because, while producing RDF is not difficult in principle, producing RDF that has the positive features of Linked Data (like “Cool URIs”) and well-planned predicates is not trivial. Therefore, discussion centered on whether there are useful improvements that can be made in advance of any “wholesale” switch to RDF generation. We considered whether communication between Cytoscape [22,23], the data warehouse systems BioMart and InterMine, and the workflow design tool Galaxy, could be made more robust and lower maintenance. It was felt that the current situation could be improved by avoiding bespoke plugins for communication. For Galaxy [101,102], the user is responsible for making sure the data is of the correct type and format for the analysis being performed. For communication between Galaxy and BioMart neither side deals with semantics yet, but generally interoperation was considered to be sufficient. This was also the case for interoperation between InterMine-based systems and Galaxy. For Cytoscape and BioMart interoperation, the existing REST API was deemed sufficient for data retrieval. We now detail the various new and changed features in these various tools.

Individual BioMart deployers decide on their own meta-data layer, and the BioMart Graphical User Interface (GUI) uses this layer to plan/construct queries, rather than the data itself. Although the GUI provides a number of options, there is nothing preventing a deployer from using non-standard names. Thus, to achieve interoperation between different BioMart instances, it is essential to harmonize the semantics used by each deployer. For example, if two BioMart instances call a data field a “UniProt identifier”, it is the responsibility of the two deployers to confirm that they are, in fact, referring to the same data-type values. As a lightweight solution, the Hackathon attendees suggested that it would be useful to have a controlled name-space for such use and/or a hand-shake between integrated instances to check that matching values are being used. Similarly, instances of the InterMine warehouse can ask each other what data they provide but do not confirm that the name-spaces are compatible. It would be beneficial if this could be checked automatically. Likewise, interoperation would be easier if the InterMine and BioMart could automatically discover the data that are provided by any given warehouse instance. It was agreed that it would help to have a more formal description of data and services and this would make planned interoperation between InterMine and BioMart easier to implement. One simple improvement would be passing headers with data column descriptions. Experience at the Ontario Institute for Cancer Research (OICR) suggests that available data-describing controlled vocabularies are rather limited. As such, a move towards Semantic Web technologies in the near future would be extremely beneficial to these two projects.

### Data exchange

#### File formats

The preceding section demonstrates that agreement on semantics is critical regardless of the use of formal Semantic Web technologies for representation and exchange of information. This is useful even at the relatively primitive level of using consistent terms to describe different file formats. For instance, the Open Bio* projects (BioPerl, Biopython, BioRuby, BioJava and EMBOSS) have effectively agreed to a file-format naming system covering major sequence formats, alignment formats, and assorted tool output formats. Similarly the XML descriptions of different tools in Galaxy describe valid input formats and the generated output formats by string based names.

It was felt that, to facilitate automated interoperability, it would be useful to have a common machine-readable namespace that allows one to assert something is a particular file format. However, agreeing on shared terms for different file formats is still only a first-step; the content of that file can also influence how a piece of software might need to operate on it. For example, the FASTA format can represent quite diverse data: nucleotide vs protein sequences, single records vs multiple records, use of ambiguity codes or not, soft masked vs plain sequence, and gapped vs ungapped. Using a more extensive and formal naming scheme able to capture these kinds of details, such as a URI, would enable the encapsulation of meta-data about the type of file and its contents, enabling validation and providing a means to prevent inappropriate actions such as using protein queries for a nucleotide sequence search. Likewise, if a set of records from two sources were received, it would be possible to ensure that they were conforming to the same representation as well as to the same biological entity. The EDAM ontology (described below) was selected as an appropriate annotation vocabulary for this purpose.

#### File formats vs. labeling of data itself

As mentioned above, combined analysis of separate data sources involves cross-referencing record fields whose names are under the control of individual data providers, and therefore may not be shared in common, or may be shared, but used to refer to different data entities. To alleviate this, BioMart and InterMine agreed to begin comparing their database column-headings to find those with a common intent, and work jointly towards a common name. For example, both provide data headed "GO identifier", but it is also necessary to assert, to software, that the columns from the two sources are truly equivalent. A URI-fragment was chosen as the solution to this problem; the URI-fragment indicates the namespace of data in that column, and when concatenated with the identifier in that column, becomes the URI of the entity in that column cell. Columns between two datasets can then be compared based on these URI-fragments. The URIs used to label columns were chosen from the EBI Ontology Lookup Service (OLS) [103]. For instance, http://www.ebi.ac.uk/ontology-lookup/?termId=MI%3A0448 provides an identifier, MI:0448, for Gene Ontology. Consideration was given to the need for a central naming authority/namespace provider. As well as the OLS, RDF data is provided by UniProt, and thus the EBI and corresponding institutes in Japan (DDBJ) and the US (NCBI) were considered natural coordinators for this role. It was further agreed that these decisions would not be imposed on individual providers, but that all providers would be encouraged to make such a migration as quickly as possible.

#### Versioning of data

A related point was that of being explicit about which version of the data is being referred to in a given RDF triple (e.g., the genome build/annotation release). For example, there are extant issues with public resources being generated from specific, now legacy, versions of genome datasets, e.g., Affymetrix microarrays. It is neither easy, nor desirable to enforce the use of just one version of the genome and the corresponding updating of older resources, and therefore there must be some clear way of tracking URIs relating to the same, re-versioned entity.

Ensembl [104,105] has addressed this problem for all of its hosted genomes, where the mapping of their versions to similar resources hosted by themselves and others (e.g., from UCSC [106] and between Ensembl releases) are systemized. A combination of the assembly version and Ensembl gene-build version are sufficient to resolve all ambiguities. Therefore, it was discussed that, when minting URIs for an RDF representation of Ensembl data, the URI schema would be based on Ensembl genome/annotation versions until another approach was deemed necessary. In addition, these URIs would be associated to other versions (both local and remote) using an appropriately named predicate.

modMine [107], an InterMine instance that is the central data integration point within the modENCODE project, records the genome version against which data were generated. However, at the time of the BioHackathon, exported data did not include the genome version, and it was therefore recommended that genome version information be included in all exported data.

Galaxy preserves data provided for each run of a Galaxy workflow. This is useful with respect to reproducibility and clarity about the analysis that was done on any given day, even over the course of a multi-year project. However, while Galaxy encourages users to record important metadata such as the genome build version, it does not enforce this. As such, users should be aware that it is their responsibility, even in Galaxy’s metadata-preserving environment, to provide the metadata critical to comprehensively describing the experiments they are running using the Galaxy tool.

Finally, BioMart and the UCSC genome browser provide version information, but they do not use the same namespace to describe this version information in every case. Thus, it can be difficult to automatically determine that two datasets are from the same version/build.

#### Data exchange conclusions

• Current systems are arbitrary but in fact can be made to work with close coordination of the development teams. This, however, is an unusual case that may only be applicable in a BioHackathon situation. Moreover, other software will have to engage in a similar re-engineering process to use these data sources.

• A namespace for file formats would be useful - candidates include the EDAM (EMBRACE Data and Methods) ontology [108] and myGrid-Moby Web service ontologies [109], which lists several of the more popular formats (e.g., GenBank flatfile) and could be extended with additional formats over time (by the time of this writing, the myGrid-Moby data-type ontology has been non-redundantly imported into the appropriate portion of the EDAM ontology, so this dichotomous choice no longer exists, and EDAM should be considered canonical in preference to the BioMOBY ontologies).

• A namespace for columns of tabular data would be useful. This utility could also be extended to describe data in more generic formats such as XML, though this was not deemed necessary at this time.

• At the BioHackathon there was general agreement among these sub-group members that the identification of the data column itself was more immediately important for data exchange and interoperability than assigning URIs to each data element in that column; however the latter seems plausible to automate if the column headers are themselves URIs. Shared, human-readable column names, however, provide a temporary solution to this integration problem through specific engineering of the client software to recognize and respond to these names correctly.

#### RDF for interoperation

If all resources expressed their data as RDF, using a shared URI scheme for third-party data elements, large-scale data integration would be dramatically facilitated, whether that be a conventional warehouse or a triple store. This, however, leads to the barrier discussed earlier relating to agreement on URI schemes, and how to accommodate RDF-linking to data from third-parties who have not yet made a commitment to either RDF or even stable URIs. In practice, RDF warehouses (such as Bio2RDF) are making these decisions on their own, and (effectively) imposing their own URIs schemes on data elements that they do not “own”. Source providers, however, find this objectionable, and even in the context of the BioHackathon community, would not agree to use these imposed URIs as their own URIs, even in cases where they did not publish stable URIs. This will lead to quite serious conflicts when the individual data providers begin publishing their own RDF, since statements (triples) will have been made throughout the Semantic Web that involve the non-canonical URIs coming from the RDF warehouses. Each of these non-canonical statements will then have to be explicitly mapped to the provider-published URI at some later point in time - a task that is effectively impossible to achieve.

This remains a difficult problem to overcome since at its core is the different pressures of need versus resourcing. For instance, the modENCODE and OICR Cancer Genome projects, represented at the BioHackathon, provide original data and therefore could generate their own canonical URIs; however, the pressures of delivering for their target communities specific needs interferes with their desire to transition to RDF publishing, due to both the additional infrastructure required, as well as the additional rigor that RDF publishing can, when done with care, necessitate. As such, this BioHackathon group suggested that a balance was needed between cleanliness/rigor and making the data available immediately in RDF. Nevertheless, they recognized that an advantage of generating RDF was that it forced producers to think more clearly about how they defined their data elements, and that this was of long-term benefit to both the providers themselves as well as the community.

Care is still needed, however, since “low quality” or unstable RDF can severely damage interoperability long-term; RDF is specifically intended to be built-upon and extended by third-parties on the Semantic Web. When dramatic changes are made to either the URIs or the nature of the statements being made about them, contradictory, even nonsensical information can result. For example, if the provider first publishes a URI that represents a gene locus record, and later realizes that this URI more accurately represents a specific Open Reading Frame (ORF), third party statements made about that URI as a locus will now resolve to statements about that ORF. Providers are cautioned, however, that these semantic shifts can also be quite subtle. For example, when a provider publishes a URI representing a protein (which might be the source of a protein-binding interaction triple in RDF) and then changes the meaning of that URI such that it now represents the *database record* for a protein (which might be the subject of triples about authorship, editorial changes, record-length, etc.), a Semantic Web agent can make extremely troublesome data integration errors. Since on the traditional Web, these semantic differences are never taken into account (because humans can automatically make the distinctions), publishing RDF requires additional care on the part of the provider compared to traditional Web publishing.

### Tools and clients for analysis and visualization

#### Open Bio*

A rare opportunity provided by BioHackathon 2010 was that representatives from the BioRuby, Biopython, BioPerl and BioJava communities were in attendance with a common goal - the production and consumption of RDF and associated technologies. An initial survey among different languages reported that the core RDF data format is very well supported by Java, Perl, Python, and Ruby libraries (Table 1). Considering RDF as a data model used mainly in the back-end for building a knowledge base, or in the middleware for data exchange, the priority of Open Bio* projects is the capacity to consume SPARQL endpoints easily, providing support to the end user for a better experience. To achieve this goal and for better uniformity, the BioHackathon encourages development of a set of shared APIs among the Open Bio* projects.

**Table 1:** RDF/SPARQL libraries for each programming language

**Language**	**Library**	**Web site**
Ruby	ActiveRDF, RDF.rb	http://activerdf.org/,http://ruby-rdf.github.com/rdf/
Java	Jena	http://jena.apache.org/
Python	RDFLib	http://www.rdflib.net/
Perl	PerlRDF	http://www.perlrdf.org/

With respect to supporting SPARQL endpoints, query systems such as the one provided by BioMart and FlyMine were considered good starting points from the perspective of interface behavior. The notable thing in these systems is that the user can explore the data/knowledge while dynamically building the result set. Using a similar approach, it was proposed that Open Bio* projects should access individual SPARQL endpoints with the goal of dynamically generating cross-domain queries fragment-by-fragment. This design, known as a “Builder Pattern”, accumulates filters and attributes stepwise (e.g., “add_filter” as an API method) during exploration of large, dispersed data-sets such as BioMart, InterMine, and Bio2RDF; this object can then be converted into one or more SPARQL queries to reproduce the exploration process in its entirety. The Biopython and BioRuby projects have implemented versions of such an interface, and others are under development.

As the number of SPARQL endpoints grows, such interfaces will become increasingly useful. The approach of providing an object-oriented adaptor API on top of SPARQL endpoints, which has a similar “look and feel” to other OpenBio* project APIs, eases the transition to using Semantic data. Current limitations are, not unexpectedly, that custom code must be written for endpoints that do not use shared ontologies and graph structures, and that currently the system does not generalize to arbitrarily complex SPARQL queries. Nevertheless, its primary utility is to encapsulate common query cases into a familiar interface that encourages skilled users to use and explore data provided by Semantic Web data sources.

### Visualization

This section focuses on the development of tools and methods to help the end-user - the researcher - consume data exposed over the Semantic Web. This sub-group’s BioHackathon activity was centered around Cytoscape and the development of plugins to make it possible to consume RDF data on the Cytoscape platform. Our efforts have been complemented by the development of interfaces in the G-language project [15], and discussions on the RelFinder [110,111] which is especially useful to explore locally-stored RDF data and high-performance SPARQL endpoints.

RDFScape [100] is a tool that allows the use of Cytoscape as an interface to browse and query Semantic Web knowledgebases in an interactive way. This visualization can be tuned via a variety of features, ranging from coloring or filtering information by namespace, treating datatypes as attributes, or even providing a semantic view of the content of the knowledge base, via the application of custom inference rules (Figure 2). Our sub-group’s efforts centered around the re-design of RDFScape with a reference use case from the molecular interaction session (described in the “Molecular interactions” section).

During the BioHackathon, the general robustness and the quality of the user experience was improved in the following ways: First the installation procedure has been dramatically simplified, such that RDFScape can be installed in the same way as any other Cytoscape plugin. In addition, we have re-designed the user interfaces, rationalizing the layout of commands and improving the flow of user interaction, where many actions now have an intuitive default. RDFScape provides “analysis contexts” or workspaces - pre-assembled settings for specific knowledge bases. If one of these “analysis contexts” is provided, the new user interaction flow does not require any decision to be taken from the user in order to begin exploring the knowledge base. Finally, the features of RDFScape were extended, and the user experience simplified, by providing three different RDFScape modes of operation, to be selected at start-up: 1) as an interface to query and visualize remote SPARQL endpoints, 2) to visualize and analyse a local knowledge base, and 3) for interactive analysis and reasoning on ontologies in the context of experimental data.

### User interaction

Because Semantic Web data is stored in the form of triples, a feasible interface to query the data might be to begin with a keyword, display a list of available predicates related to that keyword, and from there provide the list of possible objects. A ring interface is an effective implementation for this purpose, whereby the predicates and objects are displayed as a series of rings. Each ring contains a limited set of possible connections for the given data, shown graphically with icons.

**Figure 3:**
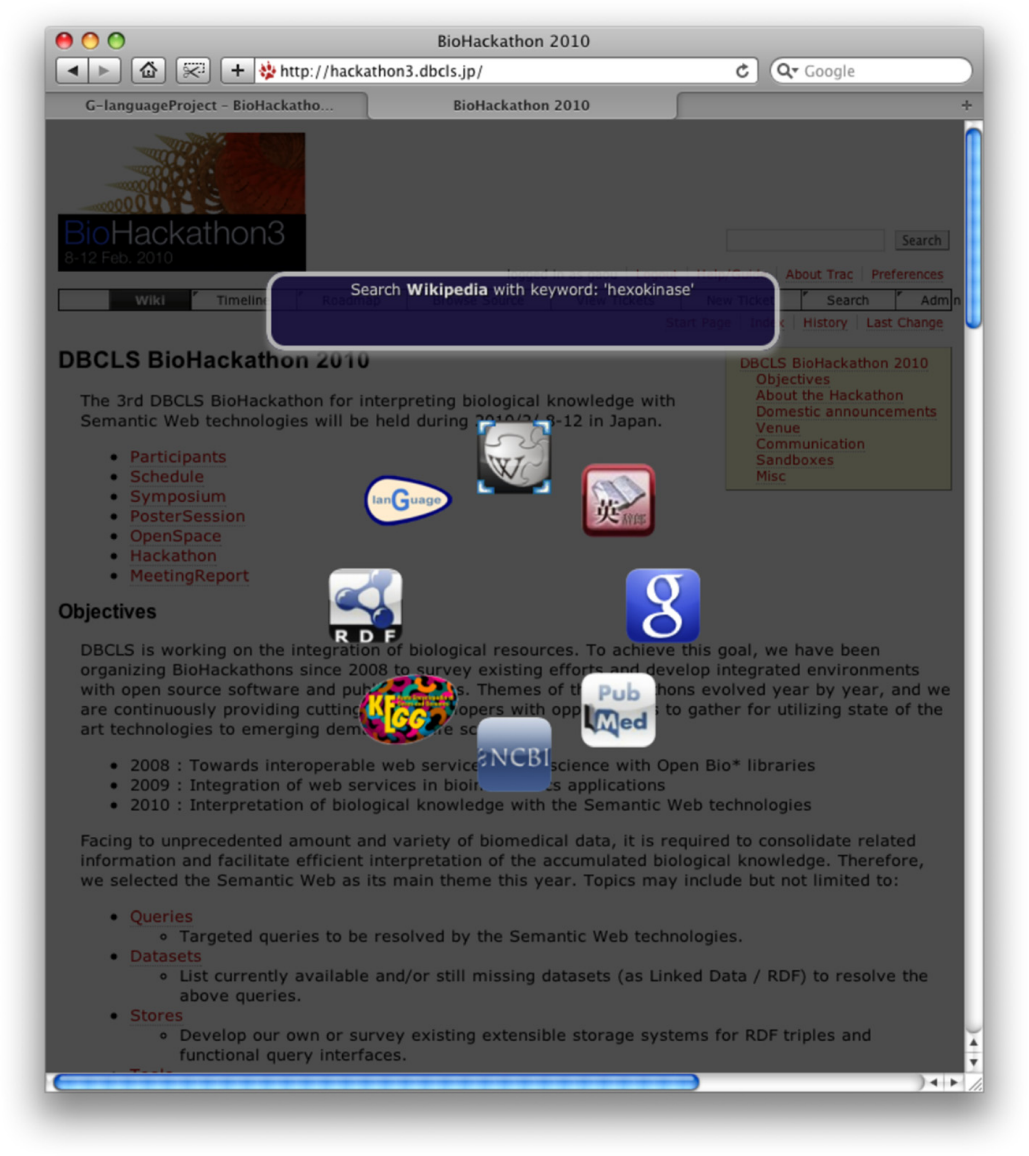
G-language Bookmarklet in action.

Searches and queries of biological data take place primarily via the Web. Therefore, a generic querying interface should be able to run on any website. Moreover, it should run without any installation of specialized software or plugins. The G-language Bookmarklet [112] is implemented as a bookmarklet coded with HTML/CSS/JavaScript, runs on almost all browsers, and can be invoked from any website. By selecting keywords of interest within any webpage and opening the G-language Bookmarklet, an array of icons in the shape of a ring appears with animation on top of the webpage that the user is currently browsing (Figure 3).

Most biological data is well-curated and cross-referenced by major database providers. An emergent Semantic Web, therefore, would complement this existing data by making the cross-references explicit and machine readable, while not disrupting the existing data publication process or existing end-user community. Using this bookmarklet, users can select the database they wish to search with the selected keyword; this is limited to frequently used websites such as Google and Wikipedia, to queries against large life-science data providers such as NCBI Entrez, EB-eye, and KEGG, and also includes SPARQL keyword-queries against Bio2RDF. Query results are displayed as a second ring of icons representing the top hits of the query; if the query returns a single match, users are redirected to the webpage of that entry, or users can mouse-select the page they wish to visit. In this way, users can take advantage of the Semantic Web and Linked Data coherently with existing data and familiar norms and paradigms for visual exploration of Web data.

### Semantic Web services

The Semantic Automated Discovery and Integration (SADI) project [113] is a set of design-patterns that allow Web services to be exposed in a manner that makes them highly compatible with other Semantic Web resources and functionalities. In the context of the BioHackathon, it served two target-audiences:

• Data providers who were uncomfortable setting up SPARQL endpoints over their entire datasets, and prefer to provide more limited access to their data and/or wish to have more fine-grained control of incoming requests over their compute resources.

• Providers of analytical tools who wish to make these tools available “transparently” as services that consume and produce Semantic Web data (i.e., RDF).

At BioHackathon 2010, the SADI project made progress on several fronts:

#### Perl SADI

We improved our documentation and developed new tutorial materials for developing SADI services in Perl. This included several updates to our “SADISeS” module on CPAN [114] that partially automates the process of building SADI Semantic Web services. In addition, we began coding compliance with the Serv ontology into our Web service support code. Identifiers that are passed out of SADI services are made to carry with them the annotations recommended by Serv, and identifiers passed into SADI services are checked to determine if they carry Serv information. Therefore, these identifiers can be used to disambiguate precisely what is being identified by the incoming URI.

#### Java SADI

Recognizing that building an OWL ontology representing a service is often the most difficult part of SADI service deployment, we undertook a project that would convert the Java class representing a service into a SADI-appropriate OWL class representing the facet restrictions of that Java class. This module, when complete, will be made available via the SADI codebase on Google Code [115] and through Maven. We also began to formulate plans to assist in deploying SADI services into the Google Cloud, though no testable outcomes were completed during the meeting.

#### Taverna SADI support

Extending our existing support for simple SADI services in Taverna, modifications were made to the Taverna engine to allow raw RDF data to be passed from service to service. This ensures that the full semantics of the data are retained, compared to moving the data representation into and out-of the internal Taverna data model. This allowed significant progress to be made towards supporting SADI Web service discovery based on complex OWL reasoning over data-type properties, which is a hallmark of the SADI system in general.

#### WSDL to SADI

Daggoo [116] is a Web service “wrapper” that learns how to use an interface by monitoring a human’s use of that interface, then explicitly encoding the semantics of that interaction to automate the “wrapping” of the Web service interface with a semantically richer framework such as BioMoby or SADI. In the case of SADI, Daggoo was enhanced such that it can read a WSDL file of a legacy Web service and provide “slots” into which data can be drag-and-dropped from the Daggoo window by the user. Since Daggoo knows the semantic type of each piece of data in the window, the user is thereby “teaching” Daggoo about the semantics of each element in the WSDL input XML schema. From there, Daggoo is able to generate automatically a mapping that exposes that WSDL service as a SADI Semantic Web service that consumes RDF and automatically converts that to an appropriate XML.

## Conclusions

BioHackathon 2010 was the one of the earliest meetings in the life sciences assembling such a large group of data providers, service developers and application projects to encourage database providers to produce their resources as Linked Data. For this purpose, we explored and tested guidelines on minting standardized URIs and predicates for creating interoperable RDF data, and observed how difficult it is to achieve consensus even within a highly collaborative community such as the BioHackathon. Means for introducing semantics into messages shared among existing biological applications were explored, which led to practical short-term solutions and long-term objectives, including the use of RDF with shared ontologies, and the role of RDF in this task became clearer. Furthermore, to fully utilize these RDF data in analytical workflows, further efforts were made to support native RDF-consuming Web services. At every step, we noted that it was necessary for providers to think about defining data very clearly with an eye to the long-term use and benefit to the community; unlike traditional Web data, RDF data is (a) meant to be automatically shared, and (b) meant to be extended by third-parties. The consequence is that RDF is more sensitive to subtle changes in intent - hence being called a ‘semantic’ technology.

### Future directions

A variety of other relevant topics were raised at the BioHackathon, but not extensively addressed. We include these here to list some open issues that might be pursued by the wider bioinformatics community, as well as by BioHackers.

• For many applications of relations in biological research it is crucial to be able to make statements regarding the origin of these relations (e.g., 'experimental' versus 'computationally inferred'). For this purpose, an initial ontology named 'evidence codes' has been implemented [117]. A further development of this is the provision of typed numerical confidence values for relations (e.g., the confidence for mouse gene A being an ortholog of human gene B is of type probability and has a value of 0.9). The providers present at BioHackathon discussed several additional open issues where a ready-to-implement consensus still needs some future work.

• Distinguishing *entities*, the biological thing or concept, from *entries*, the data provider's record of the entity. This is needed to separate properly the provenance information from the biological one, preventing reasoners and other means of automated knowledge discovery from drawing wrong conclusions.

• Sharing predicates: to relate pieces of information from different data sets to each other, data sets should share as many predicates as possible. Means of simplifying this must be found such that it is easier for RDF authors to (correctly) re-use existing predicates than to create their own. This is particularly important in the context of projects such as SADI that rely extensively on logical reasoning to achieve their integrative behaviors. Predicate re-use helps ensure that the maximum number of data services can be discovered, thus enhancing the user’s ability to automatically analyze their data.

• Versioning and updates: to make statements unambiguous and results reproducible, entries should be referred to by their specific version. Currently, not all providers implement versioned data. This requirement could be addressed with named graphs, or by relating different versions to each other with predicates, but there needs to be additional consideration by the community around this important issue, and some consensus reached as quickly as possible to minimize the impact of changes.

• Temporal knowledge representation: the notion of time is an important component in life sciences (transcriptomics, for instance). Various ontologies deal with the issue of time, but do so differently. Some effort should be spent in building a common model for use-cases (e.g., the varying treatment regime, and symptomatology, of an admitted patient over the course of their hospitalization, and how these relate to one another temporally).

• Capturing non-crisp biological knowledge: biological facts are commonly reported in fuzzy sentences by employing various adverbs (e.g., protein sar1 is *usually* located in the nuclear membrane).

• Integration of multimedia: images and videos are common ways of capturing and explaining biological facts. This may be done via semantic tagging for instance.

• Finally, the lack of user-friendly interfaces to access integrated systems is still a hurdle for many end users (consumers), and even providers. This reduces the motivation of the community of providers because, while integration is facilitated in general, and larger datasets can be created more quickly and at lower cost, making these integrated datasets accessible to their end-users is an even greater challenge than previously.

The next challenge for our community, and therefore the theme of coming BioHackathons, is to deliver this technology to the end-users in a way that maximizes the power of semantic representations, yet does not make the user responsible for understanding these additional complexities.

## Abbreviations

API: Application Programming Interface; BioPAX: Biological Pathway Exchange; CBRC: Computational Biology Research Center; CDAO: Comparative Data Analysis Ontology; CSS: Cascading Style Sheet; DBCLS: Database Center for Life Science; DDBJ: DNA Data Bank of Japan; EBI: European Bioinformatics Institute; EDAM: EMBRACE Data And Methods; FASTA: Fast Alignment; FTP: File Transfer Protocol; GTPS: Gene Trek in Prokaryote Space; GUI: Graphical User Interface; HCLSIG: Semantic Web Healthcare and Life Science Interest Group; HTML: Hypertext Markup Language; HTTP: Hypertext Transfer Protocol; HUPO: Human Proteome Organization; INSD: International Nucleotide Sequence Database; INSDC: International Nucleotide Sequence Database Collaboration; KEGG: Kyoto Encyclopedia of Genes and Genomes; LODD: Linked Open Drug Data; LSRN: Life Science Resource Names; MINT: Molecular Interaction Database; mmCIF: Macromolecular Crystallographic Information File; MPIDB: Microbial Protein Interaction Database; N3: Notation 3; NCBI: National Center for Biotechnology Information; OICR: Ontario Institute for Cancer Research; OLS: Ontology Lookup Service; OWL: Web Ontology Language; PDBj: Protein Data Bank Japan; PDBML: Protein Data Bank Markup Language; PSI-MI: Proteomics Standards Initiative Molecular Interaction; PSICQUIC: Proteomics Standard Initiative Common QUery InterfaCe; RDF: Resource Description Framework; RDFa: RDF Annotations; SPARQL: SPARQL Protocol And RDF Query Language; SADI: Semantic Automated Discovery and Integration; SBML: Systems Biology Markup Language; SIO: Semanticscience Integrated Ontology; TDWG: Taxonomic Database Working Group; TogoWS: Togo Web service; TogoDB: Togo Database; URIs: Uniform Resource Identifiers; W3C: World Wide Web Consortium; WSDL: Web Services Description Language; XML: Extensible Markup Language; XSL: XML Schema Language.

## Competing interests

The authors declare that they have no competing interests.

## Authors’ contributions

All authors attended the BioHackathon 2010. TK, MDW, and GM primarily wrote the manuscript. TK, SK, AY, MN, YY, SO, KO, HC, and TT organized the BioHackathon 2010. All authors read and approved the final manuscript.
